# Institutional roles, structures, funding and research partnerships towards evidence-informed policy-making: a multisector survey among policy-makers in Nigeria

**DOI:** 10.1186/s12961-023-00971-1

**Published:** 2023-05-26

**Authors:** Chigozie Jesse Uneke, Ijeoma Nkem Okedo‑Alex, Ifeyinwa Chizoba Akamike, Bilikis Iyabo Uneke, Irene Ifeyinwa Eze, Onyekachi Echefu Chukwu, Kingsley Igboji Otubo, Henry C. Urochukwu

**Affiliations:** African Institute for Health Policy and Health Systems, Ebonyi State University (EBSU), Abakaliki, Nigeria

**Keywords:** Institutional roles, Funding, Research partnerships, Evidence-informed policy-making

## Abstract

**Background:**

Evidence-informed policy-making aims to ensure that the best and most relevant evidence is systematically generated and used for policy-making. The aim of this study was to assess institutional structures, funding, policy-maker perspectives on researcher–policy-maker interactions and the use of research evidence in policy-making in five states in Nigeria.

**Methods:**

This was a cross-sectional study carried out among 209 participants from two geopolitical zones in Nigeria. Study participants included programme officers/secretaries, managers/department/facility heads and state coordinators/directors/presidents/chairpersons in various ministries and the National Assembly. A pretested semi-structured self-administered questionnaire on a five-point Likert scale was used to collect information on institutional structures for policy and policy-making in participants’ organizations, the use of research evidence in policy and policy-making processes, and the status of funding for policy-relevant research in the participants’ organizations. Data were analysed using IBM SPSS version 20 software.

**Results:**

The majority of the respondents were older than 45 years (73.2%), were male (63.2) and had spent 5 years or less (74.6%) in their present position. The majority of the respondents’ organizations had a policy in place on research involving all key stakeholders (63.6%), integration of stakeholders’ views within the policy on research (58.9%) and a forum to coordinate the setting of research priorities (61.2%). A high mean score of 3.26 was found for the use of routine data generated from within the participants’ organizations. Funding for policy-relevant research was captured in the budget (mean = 3.47) but was inadequate (mean = 2.53) and mostly donor-driven (mean = 3.64). Funding approval and release/access processes were also reported to be cumbersome, with mean scores of 3.74 and 3.89, respectively. The results showed that capacity existed among career policy-makers and the Department of Planning, Research and Statistics to advocate for internal funds (mean = 3.55) and to attract external funds such as grants (3.76) for policy-relevant research. Interaction as part of the priority-setting process (mean = 3.01) was the most highly rated form of policy-maker–researcher interaction, while long-term partnerships with researchers (mean = 2.61) had the lower mean score. The agreement that involving policy-makers in the planning and execution of programmes could enhance the evidence-to-policy process had the highest score (mean = 4.40).

**Conclusion:**

The study revealed that although institutional structures such as institutional policies, fora and stakeholder engagement existed in the organizations studied, there was suboptimal use of evidence obtained from research initiated by both internal and external researchers. Organizations surveyed had budget lines for research, but this funding was depicted as inadequate. There was suboptimal actual participation of policy-makers in the co-creation, production and dissemination of evidence. The implementation of contextually relevant and sustained mutual institutional policy-maker–researcher engagement approaches is needed to promote evidence-informed policy-making. Thus there is a need for institutional prioritization and commitment to research evidence generation.

## Background

There has been a growing focus on the use of quality evidence in policy-making because policies based on sound scientific processes are more likely to be successfully implemented with sustainable outcomes [1, 2]. This is especially so when there is alignment with other personal and political factors which play significant roles in policy-making [3]. Evidence-informed policy-making (EIPM) is defined as an approach in which quality available research evidence informs policy- and decision-making [4].

EIPM has become invaluable in policy-making, and there is now worldwide recognition of EIPM as a key strategy for strengthening health systems and for the overall improvement of health outcomes because of its important value in the policy-making process [5, 6]. There has been advocacy for an increase in the development and implementation of policies that are informed by evidence [1, 2]. According to WHO, when robust evidence is used in the formulation of policies, such policies have a higher likelihood of successful implementation and potential for saving more lives, utilizing scarce resources more efficiently and better meeting citizens' needs [7]. This explains why, beyond the health sector, EIPM is also increasingly gaining momentum in other critical sectors of society including agriculture [8, 9], environment [10, 11], education [12–14] and government [15].

It must be noted that the use of evidence in policy-making is complicated. The process is further compounded by the myriad definitions of what evidence actually is and the fact that it is highly content- and context-specific [16]. A good definition of evidence is provided by Strydom and colleagues as either scientific (research/surveys, quantitative/statistical data, qualitative data) or colloquial (economic, attitudinal, behavioural and anecdotal, expert opinion, propaganda, judgements, insight/experience, history, analogies, local knowledge and culture) [17].

As part of the recipe for effective use of evidence in policy-making, ingredients such as institutional skills and capacity development, leadership and governance, administrative structures and strong mutual researcher–policy-maker relationships are required [18, 19]. Thus, both individuals and institutions have key roles to play in evidence generation and use, and are overall influenced by the environment in which they are located. The individual-level role considers skills, experience, confidence and motivation, while organizational structure roles refer to systems and processes such as vision, funding, leadership, human resources and infrastructure [18, 20]. In developing countries such as Nigeria, gaps still exist in the adequacy of institutional capacity for implementing and supporting EIPM [20].

Policy-maker–researcher interactions have increasingly been recognized as critical in translating evidence to policy [21]. Adequate and meaningful contact between researchers and policy-makers is necessary to bridge the gap between research and decision-making, thereby contributing to health and social systems strengthening [22]. Partnerships between researchers and policy-makers have been found to be suboptimal, with many identified barriers to effective knowledge translation and use of research evidence in decision-making by policy-makers. [21, 23, 24].

Health and non-health institutions need to enshrine evidence in decision-making to achieve sustainable development. Although some progress has been made in defining and understanding the importance of EIPM, gaps still exist in the institutional use of evidence, especially in low- and middle-income countries (LMICs). These include a lack of locally relevant evidence, poor presentation of research findings, and low institutional prioritization of evidence use [20]. Other constraints to the institutional use of evidence for policy-making include poor demand, inadequate capacity for conducting policy-relevant research, poor budgetary allocation, lack of researcher–policy-maker interactions and limited dissemination of research evidence to policy-makers [25]. These gaps expose the burgeoning inherent weaknesses of institutional mechanisms and structures for evidence use.

Institutional structures for effective use of evidence include but are not limited to human research capacity, policy documents on research and evidence use, designated funding, incentives, research leadership and governance, logistical demands such as power supply, internet access, equipment (laptops, offices) and external partnership/support from research institutions [20, 25–28]. Nigeria is one of the LMICs where the importance of EIPM is being promoted and gradually being acknowledged among various government ministries [29–31]. However, the lack of sufficient information on the institutional roles, structures, funding and research partnerships for effective use of evidence is a major obstacle in the development of EIPM among government ministries. The need to use evidence for decision-making at the subnational and lower levels cannot be overemphasized, because these levels often make their own policies and/or adapt and contextualize higher-level (national/international) policies. The state ministries, departments and agencies (MDA) mirror the institutional arrangements of their federal counterparts. In each MDA, the Department of Planning, Research and Statistics (DPRS) or its equivalent has critical roles to play in evidence generation and translation into policy. For instance, the Federal Ministry of Health (FMOH) provides oversight and technical assistance to the state ministries of health. Its Department of Health Planning, Research and Statistics (DHPRS) has a Research, Knowledge and Management division and Policy and Planning division devoted to evidence and policy generation, respectively. This arrangement is duplicated in the state ministries of health with varying forms of data sharing and collaboration, with the FMOH DHPRS as part of the national health information management system [32]. As expected, the state-level data and research are directly relevant to the populations they serve and are used for decision-making at the state level. Thus there is a strong need to strengthen structures and capacity for evidence-based decision-making at the state level. The purpose of this study was to assess institutional roles, structures, funding and research partnerships with regard to evidence use in policy-making among various government ministries in five states in Nigeria as part of the effort to strengthen the EIPM process in the country.

## Methods

### Description of study area

There are six geopolitical zones in Nigeria, namely North Central, North East, North West, South East, South-South and South West. This study was conducted in states located in two of these six zones: Ebonyi and Enugu in the South East, and Abuja, Plateau and Kogi in the North Central zone. The country is diverse geographically, with climate ranging from arid to humid equatorial. The country also has a diversity of tribes with hundreds of languages, including Yoruba, Igbo, Hausa, Fula, Edo, Ibibio, Tiv and others. English is Nigeria's lingua franca and also the language of academic instruction [33]. A federal system of government is practised, with each state responsible for its own policies, but usually adapted from the national policy documents with respect to each sector.

### Study population/design

Participants included programme officers/secretaries, managers, heads of departments, facility heads, state coordinators and directors in various ministries and the National Assembly. This study employed a cross-sectional design. A total of 209 participants were purposively selected from various ministries (Health, Education, Environment, Agriculture, Budget & Planning, Women Affairs, Local Government) and the National Assembly (parliament).

### Study instrument/data collection method

Data were collected over a period of 1 year from September 2020 to August 2021. Data collection was conducted by trained policy-makers who participated in a mentorship programme by the African Institute for Health Policy and Health Systems.

A pretested, semi-structured self-administered questionnaire was used for data collection. This questionnaire was adapted from two previous studies [27, 31]. Additionally, contextual knowledge including pre-study consultations with key stakeholders was used to fine-tune the study tool. The questionnaire consisted of six sections. Section A was used to collect information on the sociodemographic and other related characteristics of the respondents. Section B consisted of questions that assessed institutional structures for policy and policy-making in the participants’ organizations. Section C assessed the use of research evidence in policy and policy-making processes, and Section D assessed the status of funding for policy-relevant research in the participants’ organizations.

Section E explored the interaction/partnership with researchers and the use of research evidence for policy-making. The parameters assessed included interaction with researchers through various methods/phases: priority-setting process, co-investigation, undertaking research, supporting evidence use, acquisition of research evidence, assessing quality and applicability of evidence, presentation of research evidence to policy-makers, policy-maker–researcher interactions through legislative committees, policy dialogues, research conferences and long-term partnerships. In section F, 13 questions were used to assess individual and organizational roles in promoting EIPM. The areas investigated included the organizational ability to initiate and drive processes that facilitated collaboration and networking among stakeholders in the social sector, organizational ability to initiate and undertake political advocacy on critical issues, organizational commissioning of research and provision of incentives for research and research budgets to facilitate the uptake of evidence, the development of sustainable institutional/organizational capacity for the utilization of results in decision-making and policy implementation to improve outcomes, widespread dissemination of research results and feedback from policy-makers, and the introduction of effective policy monitoring and evaluation (M&E) mechanisms to enhance the evidence-to-policy process.

Three questions with “yes” and “no” options were used to assess institutional structures for policy and policy-making in participants’ organizations. The use of research evidence in policy and policy-making processes was assessed using four questions on a five-point Likert scale with options ranging from 1 = grossly inadequate to 5 = very adequate. Funding for policy-relevant research was assessed using eight questions on a five-point Likert scale with options ranging from 1 = strongly disagree to 5 = strongly agree. The interaction/partnership with researchers and use of research evidence for policy-making was assessed using a five-point Likert scale score as follows: 1 = never, 2 = rarely, 3 = occasionally, 4 = frequently, 5 = very frequently. The questions on individual and organizational roles in promoting EIPM were evaluated using a five-point Likert scale graded as 1 = strongly agree, 2 = disagree, 3 = indifferent, 4 = agree and 5 = strongly agree.

### Theoretical framework

This study is based on the COM-B (Capability, Opportunity, Motivation—Behaviour) model developed by Michie and colleagues [34]. The model proposes that capability, opportunity and motivation are the three mechanisms that mediate human behaviour change and are in turn influenced by behaviour. These three factors are also called components.

Capability refers to the psychological and physical capacity to engage in a specified activity (knowledge and skills). Opportunity encompasses all factors external to the individual that increase the possibility of the behaviour or stimulate it. Motivation is defined as those automatic and reflective mental processes that promote and determine behaviour. The COM-B model forms the core of the behaviour change wheel (BCW) which proposes a set of nine interventions that address the gaps in capability, opportunity or motivation. Furthermore, the wheel further expands into seven policy options that can facilitate these interventions [34]. See Appendix 1 for the application of COM-B theory to the classification of institutional roles, structures, funding and research partnerships as used in this study. It should be noted that this study mostly assessed institutions rather than individuals and thus had limited or no categories under the capability and motivation components of the COM-B theory, as both assess individual-level factors.

### Data management and analysis

Data were analysed using the IBM SPSS version 20 software package. Frequencies and proportions were calculated for categorical variables. The independent variables were the sociodemographic and other related characteristics of the respondents such as age, gender, location, designation, duration in position, level of operation in organization and level of influence on policy-makers.

## Results

Table 1 shows that the majority of respondents were older than 45 years (73.2%) and had spent 5 years or less (74.6%) in their present position. The Ministry of Health had the highest number of representatives (44%). Only the Northern and Eastern regions of the country were represented. More than half of the respondents (56.9%) were state coordinators, directors, presidents or chairpersons in their institution/organization, while the majority had indirect influence on policy-making (63.6%) and operated at the state level (70.8%).

**Table 1:** Sociodemographic and work profiles of the study participants (*n* = 209)

Variable	Frequency	Percentage (%)
Age		
25–34	10	4.8
35–44	46	22.0
> 45	153	73.2
Gender		
Male	132	63.2
Female	77	36.8
Location		
Enugu	53	25.4
Abuja	61	29.2
Kogi	15	7.2
Plateau	15	7.2
Ebonyi	65	31.1
Ministry		
Ministry of Health	92	44.0
Ministry of Education	18	8.6
Ministry of Environment	1	0.5
Ministry of Agriculture	16	7.7
State government	2	1.0
Ministry of Budget and Planning	1	0.5
National Assembly	61	29.2
Ministry of Women Affairs	14	6.7
Ministry of Local Government	3	1.4
Other	1	0.5
Designation		
Programme secretaries, officers	81	38.8
Manager/department head/facility head	9	4.3
State coordinator/director/president/chairperson	119	56.9
Duration in position		
≤ 5 years	156	74.6
> 5 years	53	25.4
Influence on policy-making process		
Direct	76	36.4
Indirect	133	63.6
Level of operation in organization		
State	148	70.8
National	60	28.7
International	1	0.5

Table 2 shows that for the majority of the respondents’ organizations, a policy was in place regarding research involving all key stakeholders (63.6%), including definition and integration of stakeholders’ views within the policy on research (58.9%) and a forum to coordinate the setting of research priorities (61.2%).

**Table 2:** Institutional structures for policy and policy-making in participants’ organizations

Parameter assessed	Yes (%)	No. (%)
There is a policy on research in participant’s organization involving all key stakeholders	133 (63.6)	76 (36.4)
There is definition and integration of stakeholders’ views within a policy on research in participant’s work organization	123 (58.9)	86 (41.1)
There is a forum or process to coordinate the setting of research priorities in participant’s organization	126 (61.2)	81 (38.8)

Table 3 shows that the mean score for the use of research evidence from sources outside the participant’s organization (3.00) was similar to the mean score for use of research evidence from within the participant’s organization (2.98). A higher mean score of 3.26 was found for the use of routine data generated from within the participant’s organization. The participants rated the relevance of evidence used by their organization as high, with a mean score of 3.51.

**Table 3:** Use of research evidence in policy and policy-making processes in participants’ organizations

Parameter assessed	Mean (on a 5-point scale)
Extent to which participant’s organization uses the research done by others	3.00
Extent of use of research initiated/performed by participant’s organization for policy-making	2.98
Extent of use of data collected routinely or by survey in participant’s organization for policy-making	3.26
Relevance of evidence used by participant’s organization for policy-making	3.51

Table 4 shows that funding for policy-relevant research was captured in the budget, with a mean score of 3.47. However, this funding was reported to be inadequate (mean score: 2.53). It also shows that funding for policy-relevant research was mostly donor-driven, with a mean score of 3.64. Funding approval and release/access processes were also reported to be cumbersome, with mean scores of 3.74 and 3.89, respectively. The mean score for the existence of an M&E framework for released funds was 3.02. A high mean score was also reported for the capacity of career policy-makers and DPRS to advocate for internal funds (3.55) and attract external funds such as grants (3.76) for policy-relevant research.

**Table 4:** Funding for policy-relevant research in participants’ organizations

Parameter assessed	Mean (on 5-point scale)
Extent of agreement that funding for policy-relevant research is captured in the budget	3.47
Extent of agreement that funding for policy-relevant research is mostly donor-driven	3.64
Extent of agreement that funding for policy-relevant research is adequate	2.53
Extent of agreement that funding approval processes are cumbersome and can delay conduct of policy-relevant research	3.74
Extent of agreement that funding release and access processes are cumbersome and can delay conduct of policy-relevant research	3.89
Extent of agreement that funding released for policy-relevant research is well monitored and evaluated in order to avoid diversion and enable it to achieve its aim	3.02
Extent of agreement that career policy-makers and the Department of Planning, Research and Statistics (DPRS) unit have the capacity (e.g. memos, budget defence) to advocate/lobby for internal funds for policy-relevant research	3.55
Extent of agreement that career policy-makers and the DPRS unit have the skills and competencies to attract external funds such as grants for policy-relevant research	3.76

The highest mean scores were found for policy-maker–researcher interaction as part of the priority-setting process (mean score 3.01) and provision of assistance with undertaking research on high-priority policy issues (mean score 3.00). The lowest mean scores were found for the presence of long-term partnerships with researchers (mean score 2.61) and interaction with legislative committee testimonies and government-sponsored expert committees or public hearings (mean score 2.78) (Table 5).

**Table 5:** Interaction/partnership with researchers and use of research evidence for policy-making

Parameter assessed	Mean (on a 5-point scale)
Extent to which participant/participant’s organization interacts with researchers as part of a priority-setting process to identify high-priority policy issues for which research is needed	3.01
Extent to which participant/participant’s organization interacts with researchers as part of research about high-priority policy issues with which they were involved as a co-investigator	3.00
Extent to which participant/participant’s organization interacts with researchers to provide assistance with undertaking research about high-priority policy issues	2.81
Extent to which participant/participant’s organization interacts with researchers to provide assistance with designing and executing strategies to support policy-makers’ use of the findings from research about high-priority policy issues	2.87
Extent to which participant/participant’s organization interacts with researchers to obtain assistance with acquiring existing research evidence about high-priority policy issues	2.87
Extent to which participant/participant’s organization interacts with researchers to obtain assistance with assessing the quality and local applicability of existing research evidence about high-priority policy issues	2.86
Extent to which participant/participant’s organization interacts with researchers to obtain assistance with presenting existing research evidence about high-priority policy issues to other policy-makers in a useful way	2.84
Extent to which participant/participant’s organization interacts with researchers through legislative committee testimonies and government-sponsored expert committees or public hearings	2.78
Extent to which participant/participant’s organization interacts with researchers through policy dialogues designed to discuss high-priority policy issues and how research evidence can inform how to address these issues	2.84
Extent to which participant/participant’s organization interacts with researchers through research conferences and meetings	2.89
Extent to which participant/participant’s organization interacts with researchers through informal conversations with personal contacts on issues	2.89
Extent to which participant/participant’s organization interacts with researchers through long-term partnerships (e.g. through an advisory board) on issues	2.61

The highest mean score (4.40) was observed for the agreement that involving policy-makers in the planning and execution of programmes can enhance the evidence-to-policy process. Other highly rated parameters were the extent of agreement that the evidence-to-policy process can be enhanced if policy-makers regularly acquaint themselves with evidence produced by researchers and can also carry researchers along in the policy-making process (mean score 4.34), appointing people with proven research experience and skill into policy-making positions can enhance the evidence-to-policy process (mean score 4.34) and the institutionalization of research grants and commissioning of research by MDAs and policy-making institutions can ensure that researchers are made to focus on the core needs of policy-makers (mean score 4.34). The parameters least agreed to by the participants were that policy-relevant research is often not considered by researchers in their research works (mean score 3.08) and that mutual mistrust exists between researchers and policy-makers, and policy-makers may view research as costly, often time-consuming, and therefore a waste of resources (mean score 3.56) (Table 6).

**Table 6:** Organizational roles in promoting EIPM

Parameter assessed	Mean (on a 5-point scale)
Extent of agreement that participant’s organization can initiate and drive the process that can facilitate collaboration and networking among stakeholders in the social sector (including private-sector participants and donor agencies) to promote improved outcomes	4.14
Extent of agreement that participant’s organization can initiate and undertake political advocacy on critical issues that can ensure adequate resource mobilization (especially on how to optimize internal sources)	4.09
Extent of agreement that if the participant’s organization commissions research, provides incentives for research and research budgets, it will facilitate uptake of evidence resulting from the research for policy-making	4.09
Extent of agreement that the development of sustainable institutional/organizational capacity for the utilization of results in decision-making and policy implementation can improve outcomes	4.22
Extent of agreement that widespread dissemination of research results and feedback from policy-makers and the introduction of effective policy monitoring and evaluation mechanisms will enhance the evidence-to-policy process	4.33
Extent of agreement that the evidence-to-policy process can be enhanced if policy-makers regularly acquaint themselves with evidence produced by researchers and can also carry researchers along in the policy-making process	4.34
Extent of agreement that the joint committees and informal partnerships involving representatives of researchers, policy-makers, knowledge brokers and other stakeholders can enhance the evidence-to-policy process	4.24
Extent of agreement that mutual mistrust exists between researchers and policy-makers and that policy-makers may view research as costly, often time-consuming, and therefore a waste of resources	3.56
Extent of agreement that policy-relevant research is often not considered by researchers in their research works	3.08
Extent of agreement that there is poor communication of research findings to policy-makers	3.84
Extent of agreement that involving policy-makers in the planning and execution of research and involving researchers in the planning and execution of programmes can enhance the evidence-to-policy process	4.40
Extent of agreement that institutionalization of research grants and commissioning of research by MDAs and policy-making institutions can ensure that researchers are made to focus on the core needs of policy-makers	4.33
Extent of agreement that appointing people with proven research experience and skill to policy-making positions can enhance the evidence-to-policy process	4.34

## Discussion

This study assessed institutional structures, funding and use of research evidence in policy in five states in Nigeria. The study assessed the presence of institutional policies, fora and stakeholder engagement as some of the organizational enablers for evidence use. The results revealed that these institutional structures existed in the participants’ organizations. About two thirds of the respondents confirmed the existence of a policy on research involving all key stakeholders and that a forum or process for coordinating the setting of research priorities existed in their organizations. These can be viewed as opportunities for the generation and use of evidence in these organizations which is in accordance with the COM-B theory. Opportunities according to this theory are those factors outside the individual that can prompt a behaviour [34]. The value of engaging key stakeholders in the development of policies to create sound, transparent and trusted health policies has been documented [35].

The majority of the participants’ organizations had defined and integrated the views of stakeholders within the institutional policy on research. This finding can be seen as an advantage and sets the stage for promoting evidence use in institutional decision-making. Previous studies have identified the presence of similar institutional structures in organizations [17, 20, 36]. The findings of this study showed suboptimal use of evidence obtained from research initiated by both internal and external researchers; however, the use of routinely collected data had a high mean rating, and participants considered the evidence used in decision-making in their organization to be relevant. Institutions in LMICs tend to use more routinely collected data, with a paucity of locally initiated primary research. This could also explain why the participants considered the current evidence used to be relevant. Additionally, the weak researcher–policy-maker linkage further hampers the use of evidence from external academic institutions [24, 37]. In contrast, another study among Nigerian health policy-makers showed that the participants used survey reports and research publications external to the institution such as *Lancet* papers in the issue-raising phase of policy-making [38]. In line with bridging the research–policy gap and fostering co-production and use of evidence, it is important that organizations become more receptive to external sources of research evidence while encouraging local production. The COM-B model suggests that to improve this use of research from external sources, all three components of capability, opportunity and motivation need to be strengthened. For instance, using the BCW model, some of the intervention strategies to achieve this will include training, modelling, use of incentives and enablement [34].

Concerning funding of policy-relevant research, the organizations surveyed had budget lines for research. However, this funding was depicted as inadequate. Various studies have identified the inadequacy of funding for research as a major barrier to institutional evidence generation and use in Nigeria and other LMICs [17, 23, 26, 39]. These findings underscore the need for institutional prioritization and commitment to research evidence generation measured primarily by the amount of local funding made available for research. Funding can be seen as a form of incentivization which is a component of opportunity in the COM-B theory. Individuals or organizations can be encouraged to generate and use evidence in decision-making knowing that they have adequate financial support [34].

Donor funding was also highlighted as the major source of funding for policy-relevant research in these organizations. The critical role played by donor agencies in research evidence generation in the Global South has been highlighted in previous studies [17, 26, 40, 41]; however, there is a growing emphasis on promoting domestic funding, as this not only will encourage ownership but will also promote the use of the generated evidence for decision-making [26]. The advent and rapid progression of donor fatigue, transition, and withdrawal of funding from key health system components (including research) further accentuates the importance of alternative sources of local funding. Beyond budgeting, funding approval, release and access processes were found to be difficult and discouraged the timely execution of research projects. Availability of funding is an important form of physical opportunity for promoting behaviour as detailed in the COM-B theory developed by Michie et al. According to the BCW model, fiscal policies that encompass interventions for better funding of research activities can be used to address this [34].

It was interesting to note that the study participants considered the DPRS as having the capacity to conduct advocacy and attract both internal and external funding for research. Having the requisite knowledge, understanding and skills required to engage in a task is a vital subcomponent of capability in the COM-B theory [34]. Adequacy of institutional capacity is linked to increased evidence generation and use [42]. Therefore, this is a potential strength that needs to be harnessed in order to encourage institution-based and need-informed research. The research aspect of this key department in many public institutions in Nigeria has not been fully explored compared with its established roles in planning and statistics (generation of strategic and operational plans and routine statistics). As part of the national health research systems in the West African subregion, health research programmes in ministries of health (which is the DPRS in the Nigerian context) has been considered essential [43]. Some form of M&E for released funds was also found to exist. However, more needs to be done to promote institutional M&E of allocated funds given the marginal mean score. M&E help ensure that released funds are utilized for the programme for which they are allocated, thereby promoting accountability [44].

The study revealed inadequate interaction between researchers and policy-makers in the surveyed institutions. The avenues for these interactions such as conferences, meetings, informal communication and long-term partnerships were poorly utilized. Notably, long-term partnerships between researchers and organizations had the lowest mean scores, highlighting concerns for the sustainability of such partnerships where present. Given that partnerships such as secondment models and health advisory boards have been shown to be effective in Nigeria and similar contexts [31, 45, 46], their wide-scale implementation is highly recommended. Partnerships can be viewed as an opportunity since they provide the interaction that is needed for researchers to generate evidence relevant to the decision-makers. Although the importance of the researcher–policy-maker partnership has been well established [19, 47, 48], its implementation still falls short of expectations, as reflected in this study, possibly because of lack of awareness, motivation and enabling mechanisms for operationalization. Similar studies have also found low levels of interaction between researchers and policy-makers in Nigeria [27, 49–51].

To further demonstrate the gaps between research evidence generators and users, this study found poor partnership in the domains of co-investigation, evidence acquisition, dissemination, knowledge translation and evidence use. Active participation of decision-makers across all phases of a project including conceptualization, proposal design, field activities/implementation and result uptake afford the much-needed opportunity for collaboration and learning in order to effectively embed research in policy-making [52, 53]. Given the importance of EIPM towards sustainable development for both health and non-health sectors as surveyed, targeted strategies to promote co-conceptualization, co-creation, and co-production are needed.

Interactions with researchers in the priority-setting process had the highest mean score. Participants agreed that they interacted with researchers on high-priority issues where they were involved as co-investigators. According to the COM-B theory, such interactions which are external to the individual present the opportunity to facilitate or initiate evidence use behaviour [34]. This is most likely to have occurred in donor-funded programmes with the engagement of the ministry. Nevertheless, this does not obviate the need for more active and viable interaction models to bridge the know–do gap across all domains of policy formulation and implementation.

This study is not without limitations. Firstly, the study was based on self-reports which were not validated using other sources of evidence such as institutional reports and policy documents. However, they ensured confidentiality of their responses. Secondly, the use of only five states limits the generalizability of the findings. Nonetheless, this is one of the few studies to adopt a multisectoral approach to understanding institutional structures, funding and use of research evidence in policy-making. A large sample of policy-makers in this study is also a major strength. On the other hand, the inclusion of a multisectoral group of policy-makers from five states across two geopolitical zones is a strength of the study. Because the study was based on self-reports that were not verified by other objective means such as document reviews, the findings could be prone to social desirability bias. However, the study participants were assured of anonymity and confidentiality, which is expected to further encourage sincere responses. We acknowledge the absence of comparative analysis across the sectors included in the study as a possible limitation.

## Conclusion

This study revealed that although institutional structures such as institutional policies, fora and stakeholder engagement existed in the organizations studied, there was suboptimal use of evidence obtained from research initiated by both internal and external researchers. However, there was high use of routinely collected data, and participants considered the evidence used in decision-making in their organization to be relevant. Organizations surveyed had budget lines for research, but this funding was depicted as inadequate. Donor funding was also highlighted as the major source of funding for policy-relevant research in these organizations. There is therefore a need for institutional prioritization and commitment to research evidence generation. It is also recommended that more domestic funding be allocated to research, and approval and release of funds ensured.

## Data Availability

The datasets used and/or analysed during the current study are available from the corresponding author on reasonable request.
